# Plasma Amino Acid Concentrations at Birth and Patent Ductus Arteriosus in Very and Extremely Preterm Infants

**DOI:** 10.3389/fped.2021.647018

**Published:** 2021-02-11

**Authors:** Maurice J. Huizing, Moreyba Borges-Luján, Giacomo Cavallaro, Gema E. González-Luis, Genny Raffaeli, Pilar Bas-Suárez, Jaap A. Bakker, Rob M. Moonen, Eduardo Villamor

**Affiliations:** ^1^Department of Pediatrics, Maastricht University Medical Centre (MUMC+), School for Oncology and Developmental Biology (GROW), Maastricht, Netherlands; ^2^Department of Neonatology, Complejo Hospitalario Universitario Insular Materno-Infantil (CHUIMI) de Canarias, Las Palmas de Gran Canaria, Spain; ^3^Neonatal Intensive Care Unit, Fondazione IRCCS Cà Granda Ospedale Maggiore Policlinico, Milan, Italy; ^4^Department of Clinical Sciences and Community Health, Università degli Studi di Milano, Milan, Italy; ^5^Department of Pediatrics, Hospital Vithas Santa Catalina, Las Palmas de Gran Canaria, Spain; ^6^Department of Clinical Chemistry and Laboratory Medicine, Leiden University Medical Center, Leiden, Netherlands; ^7^Department of Pediatrics, Zuyderland Medical Center, Heerlen, Netherlands

**Keywords:** amino acids, ductus arteriosus [MeSH], glutamate, preterm (birth), metabolomic analysis

## Abstract

**Background:** Amino acids are increasingly recognized as bioactive molecules in numerous physiological and pathophysiological pathways. The non-essential amino acid glutamate is vasoactive in the rat ductus arteriosus (DA) and a decrease in its levels within the 1st days of life has been associated with the presence of patent DA (PDA) in extremely preterm infants. However, these findings have not been confirmed in other studies.

**Objective:** To investigate the possible association between amino acid concentrations in the 1st day of life and the presence of PDA in a cohort of 121 newborns with gestational age (GA) below 30 weeks and birth weight (BW) below 1,500 g.

**Methods:** Plasma samples were collected 6–12 h after birth and amino acid concentrations were determined by tandem mass spectrometry. Besides PDA, we analyzed the potential association of amino acid concentrations with infant sex, small for GA (SGA, defined as BW < third percentile), antenatal corticosteroids, chorioamnionitis, and preeclampsia. Group differences were analyzed by ANOVA adjusted for GA and BW. A Bonferroni significance threshold of *P* < 0.0024 was used to correct for multiple testing.

**Results:** PDA was found in 48 of the 121 infants examined. We observed higher mean levels of glutamate in infants with PDA (147.0 μmol/L, SD 84.0) as compared with those without (106.7 μmol/L, SD 49.1, *P* = 0.0006). None of the other amino acid concentrations in the PDA group reached the level of statistical significance that was pre-set to correct for multiple comparisons. Glutamate levels were not significantly affected by infant sex, being SGA, or by exposure to antenatal corticosteroids, clinical chorioamnionitis, or preeclampsia.

**Conclusion:** Our study not only does not confirm the previous findings of low glutamate levels in preterm infants with PDA, but we have even found elevated glutamate concentrations associated with PDA. Nevertheless, despite the high statistical significance, the difference in glutamate levels may lack clinical significance or may be an epiphenomenon associated with the particular clinical condition of infants with PDA.

## Introduction

Failed or delayed closure of the ductus arteriosus (DA) after birth leads to patent DA (PDA), a common condition among very preterm infants (1–6). The two most relevant vasoactive factors that maintain the DA open during fetal life and mediate its closure after birth are oxygen tension and prostaglandins (4, 5). However, the DA responds to numerous vasoactive agents and its closure is finally the result of a complex interaction of mediators and pathways (4, 5).

In a recent study, Fujita et al. proposed a role for the non-essential amino acid glutamate in the postnatal closure of the DA (7). They reported that plasma glutamate concentration was significantly lower in extremely preterm infants (gestational age <28 weeks) with PDA compared to those without the condition (7). In addition, they observed that intraperitoneal injection of glutamate to pregnant rats induced ductal contraction in the fetus. Moreover, they showed that the mRNA of the glutamate ionotropic receptor α-amino-3-hydroxy-5-methyl-4-isoxazolepropionate (AMPA) type subunit one was highly expressed in rat DA compared to the aorta, and was co-localized with autonomic nerve terminals in the human and rat DA. In agreement with these results, microarrays comparing the genetic profiles of the DA and ascending aorta from mice and rats showed significant changes in the ductal expression of both ionotropic and metabotropic glutamate receptors (8, 9). Altogether, these data suggest a role for glutamatergic signaling in the physiology and/or pathobiology of the DA. Nevertheless, more studies are needed to confirm this hypothesis.

Between 2007 and 2012, our group conducted a prospective study to analyze the association between polymorphisms of the *carbamoyl phosphate synthetase* (*CPS*) gene and necrotizing enterocolitis (NEC) (10–12). In that research, plasma levels of amino acids were determined in the 1st day of life in order to correlate them with the primary outcome of the study. One of the secondary outcomes of the study was PDA. Our aim was to use these unpublished data to investigate the association between glutamate, as well as other amino acids, and the presence of PDA in infants with a gestational age (GA) below 30 weeks.

## Methods

### Study Population

This study was performed in a previously described cohort of 121 preterm infants (GA ≤30 weeks and birth weight ≤1,500 g) admitted to the level III neonatal intensive care units of the Maastricht University Medical Center (Maastricht, The Netherlands), Hospital Universitario Materno-Infantil de Canarias (Las Palmas de Gran Canaria, Spain), and Carlo Poma Hospital (Mantova, Italy) between July 2007 and October 2008. All infants had been enrolled in the study “Carbamoyl Phosphate Synthetase (CPS) Polymorphisms as Risk Factor for NEC” (NCT00554866). The institutional review board (IRB) of the three participating centers approved the use of the clinical data of the patients for the present study (IRB numbers: Maastricht MEC-07-2-018; Las Palmas CEIC- 276; Mantova 21366/2007). Written informed consent was obtained from the parents for inclusion in the study. Infants exposed to a blood transfusion, enteral or parenteral proteins/amino acids, or inhaled NO before blood sampling were excluded from the study. The correlation between arginine, citrulline and dimethylarginine levels and CPS polymorphisms and NEC in the same group of patients has been the subject of another publications of our group (10, 11).

### PDA Assessment and Other Clinical Variables

The perinatal and neonatal data of enrolled infants (Table 1) were extracted from the database of the NCT00554866 trial. At the time of inclusion of the children in the cohort (2007–2008), the three participating centers had very similar protocols for detection and treatment of PDA. These protocols included the performance of a screening echocardiogram by a pediatric cardiologist on postnatal day 2 to 5. As previously described, hemodynamically significant PDA was defined by a transductal diameter >1.5 mm with unrestrictive (<1 m/s) left to right transductal flow on pulse wave Doppler and clinical signs of pulmonary overcirculation (e.g., increasing ventilation, oxygenation problems) and/or systemic hypoperfusion (e.g., abdominal distension, oliguria) (13). In the absence of clear clinical signs, ascertainment of hemodynamic significance was made on the basis of any of the following echocardiographic characteristics: left atrial/aortic root ratio >1.4, mean velocity in the left pulmonary artery >0.6 m/s, or diastolic backflow in the abdominal aorta (13).

**Table 1:** Baseline characteristics and neonatal complications in preterm infants with and without PDA.

	**PDA-yes (*n* = 48)**	***n* data missing**	**PDA-no (*n* = 73)**	***n* data missing**	***P*-value**
Birth weight (g)	967 (SD 238)	0	1,069 (SD 238)	0	0.023
Gestational age (wks)	27.5 (SD 1.5)	0	28.6 (SD 1.5)	0	0.000
Male sex	26 (54.2)	0	43 (58.9)	0	0.607
Prenatal steroids	39 (81.2)	0	55 (77.5)	2	0.159
Preeclampsia	5 (10.6)	1	20 (28.2)	2	0.023
Clinical chorioamnionitis	2 (4.2)	0	9 (12.3)	0	0.127
Vaginal delivery	16 (33.3)	0	24 (32.9)	0	0.958
Apgar (1 min)	5 (4–7)	1	7 (5–8)	0	0.079
Apgar (5 min)	8 (6–9)	1	9 (7–9)	0	0.109
RDS	36 (75.0)	0	40 (55.6)	1	0.030
Mechanical vent.	15 (83.3)	0	40 (56.3)	2	0.002
BPD	19 (45.2)	6	14 (21.2)	7	0.008
Hypotension	26 (54.2)	0	27 (37.5)	1	0.072
Sepsis	29 (63.0)	2	34 (46.6)	0	0.080
NEC	5 (10.9)	2	2 (2.9)	5	0.084
IVH	18 (37.5)	0	17 (23.6)	1	0.101
PVL	5 (10.4)	0	4 (5.5)	0	0.311
ROP	4 (10.0)	8	5 (7.5)	6	0.647
Mortality	7 (14.6)	0	9 (12.3)	0	0.720

*Results are expressed as mean (SD). median [interquartile range] or absolute numbers of patients (percentage). PDA, patent ductus arteriosus; RDS, respiratory distress syndrome; BPD, bronchopulmonary dysplasia (defined as oxygen at 36 weeks postmenstrual age); NEC, necrotizing enterocolitis (≥stage II); IVH, intraventricular hemorrhage (≥grade 2); PVL, periventricular leukomalacia; ROP, retinopathy of prematurity (≥stage II)*.

GA was determined by the last menstrual period and early ultrasounds (before 20 weeks of gestation). Besides the presence of PDA, the following demographic and clinical characteristics were collected and correlated with the amino acid concentrations: infant sex; small for GA (SGA, defined as birth weight for GA below the sex-specific third percentile); antenatal corticosteroids (defined as two doses of betamethasone administered 24 h apart); clinical chorioamnionitis (defined as every clinical suspicion of infection of the chorion, amnion, amniotic fluid, placenta, or a combination as judged by the obstetrician); and preeclampsia (based on the obstetrician's diagnosis).

### Plasma Amino Acid Analysis

One blood sample (500 μL) was obtained between 6 and 12 h after birth from an umbilical artery or peripheral artery catheter. When not available, the blood sample was obtained from venous puncture. Immediately after collection, heparinized blood samples were put on ice and centrifuged within 10 min (4,000 rpm, 10 min, 4°C) to obtain plasma. The plasma was deproteinized with 6 mg of solid 5-sulfosalicylic acid (SSA; Sigma, St. Louis, MO, USA) per 100 μL plasma, and stored at −80°C until further analysis. The samples obtained in Las Palmas and Mantova were transported on dry ice to Maastricht where all the analyses were performed (10).

Concentrations of (underivatized) amino acids in plasma were determined using an ultra-performance liquid chromatography (UPLC) separation module coupled to an electrospray ionization tandem mass spectrometry (ESI-MS/MS, Quattro Premier, Waters, Etten-Leur, The Netherlands). Stable isotope labeled amino acids were used as internal standards for quantification of plasma amino acids. All analyses were performed in one laboratory (Department of Clinical Genetics, Maastricht University Medical Center, Maastricht, The Netherlands) (10).

### Statistical Analysis

Results for continuous variables are expressed as mean (SD) or, if variables were not normally distributed, as median (interquartile range). Standardized residuals were examined for outliers, and measurements that were < -3.0 or >3.0 SD from the mean were removed. The possible relationship between amino acid concentrations and PDA was evaluated by ANOVA with adjustment for gestational age, and birth weight. Analysis included 21 ANOVA models (one clinical variable × 21 amino acid measurements) and a Bonferonni significance threshold of *P* < 0.0024 (0.05 divided by 21) was used to correct for multiple testing (14). We performed a power analysis to determine the minimum amino acid concentration ratio (PDA-yes:PDA-no) that would be reach statistical significance (*P* < 0.0024) with our sample size (15). The results of the power analysis are shown in Supplementary Table 1. All analyses were performed using IBM SPSS Statistics for Windows (Version 22.0; Armonk, NY, USA).

## Results

PDA was found in 48 of the 121 infants examined. The characteristics of the cohort are summarized in Table 1. The plasma concentrations of the different amino acids and their relation to PDA are shown in Table 2. Glutamate was the only amino acid whose concentration was significantly different (*P* < 0.0006) when the groups with and without PDA were compared. None of the clinical or perinatal characteristics, including sex, BW below third percentile, use of antenatal corticosteroids, history of chorioamnionitis, or history of preeclampsia, led to significant differences in glutamate concentrations (Table 3). Glutamate concentrations were also not significantly different in infants who developed intraventricular hemorrhage (≥grade 2), NEC (≥stage II), bronchopulmonary dysplasia (defined as supplementary oxygen at 36 weeks postmenstrual age), or retinopathy of prematurity (≥stage II) (Table 3).

**Table 2:** Plasma concentrations of amino acids (μmol/L) in preterm infants with and without PDA.

**Amino acid**	**PDA status**	**Mean**	**SD**	***N***	***P-*value**	**Adjusted *P-*value**
Glutamate	PDA-no	106.7	49.1	66	0.004	**0.0006**
	PDA-yes	147.0	84.0	47		
Asparagine	PDA-no	60.8	22.6	70	0.870	0.636
	PDA-yes	61.5	25.6	47		
Serine	PDA-no	155.8	45.3	71	0.232	0.092
	PDA-yes	168.4	61.9	48		
Glutamine	PDA-no	481.1	189.9	69	0.189	0.692
	PDA-yes	441.2	176.1	44		
Histidine	PDA-no	72.7	19.6	70	0.311	0.163
	PDA-yes	76.8	23.8	45		
Glycine	PDA-no	313.9	117.2	72	0.614	0.167
	PDA-yes	325.5	129.1	47		
Threonine	PDA-no	247.4	90.3	72	0.257	0.039
	PDA-yes	268.1	105.5	47		
Citrulline	PDA-no	21.3	6.3	68	0.894	0.807
	PDA-yes	21.2	5.5	48		
Arginine	PDA-no	45.2	22.5	73	0.026	0.074
	PDA-yes	35.7	21.9	45		
Alanine	PDA-no	329.8	178.0	69	0.718	0.441
	PDA-yes	342.2	181.5	45		
Taurine	PDA-no	208.0	94.0	73	0.664	0.468
	PDA-yes	200.9	75.6	47		
α-aminobutyric acid	PDA-no	15.9	7.6	72	0.204	0.253
	PDA-yes	18.1	10.0	48		
Tyrosine	PDA-no	109.8	31.5	69	0.015	0.027
	PDA-yes	129.7	48.8	48		
Valine	PDA-no	167.1	49.3	67	0.201	0.216
	PDA-yes	182.8	52.0	45		
Methionine	PDA-no	49.0	28.0	71	0.004	0.023
	PDA-yes	66.8	37.6	48		
Isoleucine	PDA-no	50.2	24.5	70	0.953	0.831
	PDA-yes	49.9	25.2	46		
Phenylalanine	PDA-no	68.8	15.1	70	0.606	0.358
	PDA-yes	70.7	21.2	46		
Tryptophan	PDA-no	27.7	8.2	66	0.204	0.014
	PDA-yes	29.7	7.7	46		
Leucine	PDA-no	69.1	22.7	67	0.578	0.432
	PDA-yes	71.8	27.5	44		
Ornithine	PDA-no	80.7	40.7	71	0.062	0.033
	PDA-yes	95.9	45.9	47		
Lysine	PDA-no	261.1	103.6	72	0.510	0.423
	PDA-yes	274.3	108.6	46		

*Results are expressed as mean (SD). Standardized residuals were examined for outliers, and measurements that were < -3.0 or >3.0 SD from the mean were removed. Adjusted for gestational age and birth weight. PDA, patent ductus arteriosus*.

**Table 3:** Plasma concentrations of glutamate (μmol/L) in different characteristics and outcomes of preterm infants.

**Characteristic/outcome**	**Mean**	**SD**	***N***	***P-*value**	**Adjusted *P-*value**
Female	150.3	85.4	52	0.012	0.019
Male	115.3	57.4	70		
SGA-no	128.2	74.1	101	0.523	0.857
SGA-yes	132.3	59.3	22		
Prenatal steroids-no	120.0	79.6	25	0.574	0.302
Prenatal steroids-yes	130.5	70.5	95		
Preeclampsia-no	126.6	73.7	91	0.687	0.162
Preeclampsia-yes	116.6	45.2	23		
Chorioamnionitis-no	137.1	72.5	111	0.007	0.015
Chorioamnionitis-yes	77.6	56.3	12		
BPD-no	124.4	76.6	74	0.742	0.160
BPD-yes	128.5	49.7	31		
NEC-no	129.3	70.7	105	0.196	0.224
NEC-yes	166.4	106.6	7		
IVH-no	139.4	82.8	85	0.200	0.470
IVH-yes	122.3	57.5	35		
ROP-no	122.1	63.6	93	0.157	0.066
ROP-yes	71.3	32.9	7		

*Results are expressed as mean (SD). Standardized residuals were examined for outliers, and measurements that were < -3.0 or >3.0 SD from the mean were removed. Adjusted for gestational age and birth weight. SGA, small for gestational age; BPD, bronchopulmonary dysplasia (defined as oxygen at 36 weeks postmenstrual age); NEC, necrotizing enterocolitis (≥stage II); IVH, intraventricular hemorrhage (≥grade 2); ROP, retinopathy of prematurity (≥stage II)*.

Changes in amino acid concentrations depending on infant sex, SGA, and exposure to antenatal corticosteroids, chorioamnionitis, or preeclampsia are depicted in Supplementary Tables 2–6. SGA infants showed increased plasma concentrations of asparagine, glutamine, glycine and alanine (Supplementary Table 3). Tryptophan levels were significantly reduced in infants exposed to preeclampsia (Supplementary Table 5) and α-aminobutyric acid levels were significantly increased in infants exposed to antenatal corticosteroids (Supplementary Table 6).

## Discussion

Along with their fundamental role as elements for protein and peptide synthesis, amino acids are increasingly recognized as bioactive molecules in numerous physiological and pathophysiological pathways (16). Alterations in amino acid levels during the 1st days of postnatal life have been associated with clinical conditions of prematurity such as RDS or NEC (10, 17–20). Closure of the DA at birth is one of the processes in which a participation of amino acids, specifically glutamate, has been proposed. However, data supporting this potential role of glutamate, although provocative, are still scarce (7). In the present study, we analyzed the concentrations of amino acids in plasma samples obtained between 6 and 12 h of life in a group of preterm newborns who had not yet received amino acid supplementation. Interestingly, glutamate was the only amino acid whose concentration was significantly different between the groups with and without PDA. However, in contrast to the previous report by Fujita et al. (7), glutamate levels were higher in the PDA group. None of the other amino acids showed differences in plasma concentrations in the PDA group that reached the level of statistical significance (*P* < 0.0024) preset in our analysis. In addition, neither infant sex nor obstetric or perinatal conditions significantly affected glutamate concentrations.

Glutamate is a pleiotropic molecule acting at the crossroads of metabolism and signaling, and functioning as both substrate and product in many distinct physiological processes (21–24). Glutamate is synthesized from glutamine, α-ketoglutarate and 5-oxoproline and in turn is the precursor for the biosynthesis of amino acids such as L-proline and L-arginine (24). In addition, other neurotransmitters as well as the antioxidant glutathione are also synthesized from glutamate (24). This ubiquity of glutamate and its metabolism greatly complicates the interpretation of our results. Although we have found an association between high levels of glutamate at birth and the presence of PDA, glutamate levels may be reflecting some other aspect of the perinatal clinical condition rather than having a direct effect on DA closure. Many of the maternal pathological conditions that result in preterm birth are accompanied by alterations in the aminogram that can be transferred to the fetus and affect plasma amino acid levels in the 1st h of life (25–27). However, as mentioned above, we did not find significant changes in glutamate in SGA infants or after exposure to clinical chorioamnionitis, preeclampsia, or antenatal steroids. In addition, infection and brain injury, two conditions that are very frequently present among very preterm infants, are known to alter the circulating levels of glutamate and other amino acids (28, 29).

Glutamate receptors are divided into the ligand-gated ion channel receptors (ionotropic receptors) and G protein-coupled metabotropic receptors (30). Ionotropic receptors are further classified into three groups based on their pharmacology and structural properties: *N*-methyl-D-aspartate (NMDA), AMPA and kainate receptors (30). Glutamatergic communication through NMDA receptors is key in the central nervous system where glutamate is the main excitatory neurotransmitter (21, 22). However, glutamatergic signaling may also occur in non-neuronal tissues, including the cardiovascular system (30–42).

Little is known about glutamate signaling in the vascular system even though not only endothelial and smooth muscle cells can release glutamate, but also platelets and immune cells (21, 34, 43). The vascular effects of glutamate may vary depending on the vascular bed studied. Because of its important role in nervous system homeostasis, most studies have focused on brain (micro)circulation where glutamate is a vasodilator. Nitric oxide (39), carbon monoxide (30, 40, 41), and prostaglandins (42) have been involved in glutamate-induced vasodilation in the central nervous system. As regards other vascular territories, Nguyen-Duong showed glutamate-induced relaxation in porcine coronary arteries (33). This relaxation was attenuated by 4-aminopyridine which suggests the involvement of voltage-gated K^+^ (K_V_) channels. Inhibition of K_V_ channels by reactive oxygen species is one of the main mechanisms responsible for oxygen-induced contraction of the DA (4, 5). K_V_ channel inhibition leads to membrane depolarization and increased Ca^2+^ entry through L-type Ca^2+^ channels (4, 5). Interestingly, Dumas et al. have recently reported K_V_-mediated, Ca^2+^-dependent glutamate release from human pulmonary arterial smooth muscle cells (21). Finally, as mentioned elsewhere, Fujita et al. showed glutamate-induced contraction of fetal rat DA and proposed an AMPA receptor-mediated release of noradrenaline as the mechanism underlying this contraction (7). Another interesting vascular effect of glutamate with potential impact on DA biology is its role as platelet activator, which is in part cyclooxygenase-dependent (34, 43). Platelet activation has been identified as a key factor in ductal closure (44, 45) and a potential role of glutamate might be a hypothesis to consider in future research.

The two most important limitations of our study are the relative small sample size and that is based on a single amino acid determination instead of longitudinal samples collected throughout the first days of life. In the study of Fujita et al., the sample is much smaller (16 infants) than in our study but amino acid levels were determined at birth (arterial umbilical cord blood) and on the 2nd day of life (7). Interestingly, glutamate levels were not significantly diminished in the first sample. In addition, not only glutamate but also leucine, lysine, phenylalanine, valine, tryptophan, methionine, isoleucine, arginine, tyrosine and glycine concentrations were decreased in the PDA group on the 2nd day of life (7). This was despite the fact that both the PDA and control groups received a similar regimen of amino acid supplementation. Of note, the amino acid mixture used by Fujita et al., had a much lower concentration of glutamate than the mixtures commonly used in Europe and America (7). However, the decrease in the levels of most amino acids, and not only glutamate, may suggest a general increase in the demand for amino acids in extremely preterm infants with PDA.

To the best of our knowledge, few other studies have evaluated the association between PDA and amino acid concentrations within the 1st days of life. Ryckman et al. analyzed amino acid concentrations in dried blood spot samples taken on day 1–3 in a cohort of 689 preterm infants (GA <37 weeks) of which 133 had PDA. The levels of glutamate were not affected but leucine, methionine, phenylalanine, and valine concentrations were higher in infants with PDA as compared with those without. Nevertheless, the differences did not reach the limit of statistical significance set by the authors to correct for multiple testing (*P* < 5 × 10^−5^). The authors reported similar results when the analysis was limited to the infants with GA below 32 weeks. Ryckman et al. also tested the *ex vivo* response of mouse DA to leucine, methionine, phenylalanine, and valine and observed that the amino acids failed to produce a significant change in ductal tone. Unfortunately, the ductal response to glutamate was not investigated. Very recently, Oltman et al. reported in a cohort of 34,969 infants with GA <37 weeks (1,924 infants with GA <32 weeks) that increased risk for morbidity (including PDA) or mortality was associated with increased concentrations of glycine and proline, as well as decreased concentrations of ornithine in dried blood spot samples taken during the 1st week of life (46). Finally, Bardanzellu et al. showed decreased tryptophan urinary levels (samples collected <12 h of life) in preterm infants with PDA (47). Therefore, the association between PDA and amino acid concentrations varies greatly between studies, making difficult to draw conclusions about the specific role of a particular amino acid in DA biology and pathobiology.

In summary, our study not only does not confirm the previous findings of low glutamate levels in preterm infants with PDA, but we have even found elevated glutamate concentrations associated with PDA. However, association does not mean causality. Despite the high statistical significance, the difference in glutamate levels may lack clinical significance or may be an epiphenomenon associated with the particular clinical condition of infants with PDA.

## Data Availability Statement

The raw data supporting the conclusions of this article will be made available by the authors, without undue reservation.

## Ethics Statement

The studies involving human participants were reviewed and approved by Ethics Committees from Maastricht University Medical Center (Maastricht, The Netherlands), Hospital Universitario Materno-Infantil de Canarias (Las Palmas de Gran Canaria, Spain), and Carlo Poma Hospital (Mantova, Italy). Written informed consent to participate in this study was provided by the participants' legal guardian/next of kin.

## Author Contributions

MH, MB-L, GC, GG-L, GR, and PB-S collected data for the study, contributed to interpretation of results, and reviewed and revised the manuscript. JB performed the amino acid analysis, contributed to interpretation of results, and reviewed and revised the manuscript. RM contributed to the design of the study, collected data and supervised data collection, performed the statistical analysis, contributed to interpretation of results, and reviewed and revised the manuscript. EV conceptualized the study, supervised data collection and statistical analysis, contributed to interpretation of results, and drafted the manuscript. All authors contributed to the article and approved the submitted version.

## Conflict of Interest

The authors declare that the research was conducted in the absence of any commercial or financial relationships that could be construed as a potential conflict of interest.
