# Supplementary material for: Plasma Amino Acid Concentrations at Birth and Patent Ductus Arteriosus in Very and Extremely Preterm Infants
Source: Front Pediatr. 2021 Feb 11;9:647018. doi: 10.3389/fped.2021.647018 (PMC7905031; doi:10.3389/fped.2021.647018)
Supplement: Supplementary file 1 [file Data_Sheet_1.PDF]

Plasma amino acid concentrations at birth and patent ductus arteriosus in very and extremely preterm infants

Maurice J. Huizing[1], Moreyba Borges-Luján[2], Giacomo Cavallaro[3], Gema E. González-Luis[2], Genny Raffaeli[3,4], Pilar Bas-Suárez[5], Jaap A. Bakker[6], Rob M. Moonen[7], Eduardo Villamor[1,*]

[1]Department of Pediatrics, Maastricht University Medical Centre (MUMC+), School for Oncology and Developmental Biology (GROW), Maastricht, 6202 AZ, The Netherlands.

[2]Department of Neonatology, Complejo Hospitalario Universitario Insular Materno-Infantil (CHUIMI) de Canarias, Las Palmas de Gran Canaria 35016, Spain.

[3]Neonatal Intensive Care Unit, Fondazione IRCCS Cà Granda Ospedale Maggiore Policlinico, Milan 20122, Italy.

[4]Department of Clinical Sciences and Community Health, Università degli Studi di Milano, Milan 20122, Italy

[5]Department of Pediatrics, Hospital Vithas Santa Catalina, Las Palmas de Gran Canaria, Spain.

[6]Department of Clinical Chemistry and Laboratory Medicine, Leiden University Medical Center, Leiden 2333ZA, The Netherlands.

[7]Department of Pediatrics, Zuyderland Medical Center, Heerlen 6401CX, The Netherlands.

*** Correspondence:**
Eduardo Villamor
e.villamor@mumc.nl

Supplementary Material

Supplementary Table 1. Power analysis

Amino acid	PDA yes-no ratio	Amino acid	PDA yes-no ratio
**Glutamate**	1.31	**Alpha-aminobutyric acid**	1.42
**Asparagine**	1.31	**Tyrosine**	1.23
**Serine**	1.24	**Valine**	1.20
**Glutamine**	1.30	**Methionine**	1.43
**Histidine**	1.22	**Isoleucine**	1.40
**Glycine**	1.30	**Phenylalanine**	1.20
**Threonine**	1.30	**Tryptophan**	1.17
**Citrulline**	1.21	**Leucine**	1.25
**Arginine**	1.56	**Ornithine**	1.74
**Alanine**	1.45	**Lysine**	1.30
**Taurine**	1.28		

The ratio represents minimum amino acid concentration ratio between infants with and without PDA to reach statistical significance ($P<0.0024$) with 0.80 power and a sample size of 121. PDA: patent ductus arteriosus.

Supplementary Table 2. Plasma concentrations of amino acids (µmol/L) in preterm infants: sex differences.

**Amino acid**	**Female**			**Male**			***P*-value**	***adjusted P*-value**
	**Mean (µmol/l)**	**SD**	**N**	**Mean (µmol/l)**	**SD**	**N**		
**Glutamate**	150	85	52	115	57	70	0,012	0,019
**Asparagine**	68	30	53	57	21	70	0,025	0,049
**Serine**	167	55	53	160	50	70	0,706	0,549
**Glutamine**	556	254	52	434	173	70	0,004	0,007
**Histidine**	83	31	52	72	19	71	0,024	0,025
**Glycine**	340	147	52	310	111	72	0,248	0,423
**Threonine**	248	100	51	261	87	71	0,668	0,207
**Citrulline**	21	6	53	22	5	67	0,151	0,332
**Arginine**	43	25	52	42	23	73	0,562	0,862
**Alanine**	319	182	47	345	174	71	0,323	0,272
**Taurine**	195	67	49	212	98	73	0,932	0,380
**Alpha-aminobutyric acid**	17	9	52	17	9	73	0,631	0,869
**Tyrosine**	117	43	52	124	43	73	0,527	0,407
**Valine**	173	47	47	173	50	68	0,445	0,845
**Methionine**	57	30	50	54	31	71	0,395	0,580
**Isoleucine**	59	32	53	47	23	70	0,025	0,024
**Phenylalanine**	73	22	50	68	16	71	0,368	0,130
**Tryptophan**	30	8	49	28	8	67	0,154	0,123
**Leucine**	70	21	45	70	24	69	0,152	0,999
**Ornithine**	93	48	52	85	41	71	0,649	0,460
**Lysine**	257	104	50	269	98	71	0,934	0,469

Results are expressed as mean (SD). Standardized residuals were examined for outliers, and measurements that were <−3.0 or >3.0 SD from the mean were removed. Adjusted for gestational age and birth weight.

Supplementary Table 3. Plasma concentrations of amino acids (µmol/L) in preterm infants born small for gestational age (SGA).

Amino acid	SGA- yes			SGA- no			*P*-value	*Adjusted P*-value
	**Mean (µmol/L)**	**SD**	**N**	**Mean (µmol/L)**	**SD**	**N**		
**Glutamate**	132.3	59.3	22	128.2	74.1	101	0.523	0.857
**Asparagine**	83.3	36.6	24	57.9	21.9	102	0.003	**0.00006**
**Serine**	171.3	57.5	24	162.3	56.1	103	0.587	0.585
**Glutamine**	722.3	373.5	24	445.8	166.7	100	0.001	**0.000001**
**Histidine**	75.9	26.4	23	74.4	20.1	98	0.890	0.988
**Glycine**	404.5	154.4	24	293.7	97.9	99	0.002	0.00025
**Threonine**	315.3	130.6	24	248.9	88.5	102	0.025	0.018
**Citrulline**	22.4	6.7	23	20.9	5.4	98	0.131	0.210
**Arginine**	43.5	29.0	24	42.6	23.0	103	0.997	0.824
**Alanine**	511.7	311.9	22	306.5	150.3	98	0.006	**0.00013**
**Taurine**	174.7	66.1	22	214.7	92.3	103	0.308	0.122
**alpha-Aminobutyric acid**	18.1	10.6	24	16.4	7.9	103	0.543	0.271
**Tyrosine**	118.5	47.8	24	115.9	35.8	98	0.707	0.653
**Valine**	146.6	31.1	19	183.4	55.8	100	0.000	0.011
**Methionine**	57.6	30.0	23	54.7	32.2	101	0.647	0.283
**Isoleucine**	41.8	25.1	23	52.4	24.6	98	0.066	0.028
**Phenylalanine**	71.5	26.1	22	70.0	17.3	101	0.386	0.879
**Tryptophan**	28.5	8.3	24	30.6	10.3	100	0.189	0.064
**Leucine**	60.6	15.2	19	71.4	23.7	96	0.059	0.046
**Ornithine**	95.7	53.6	24	84.0	38.1	99	0.776	0.269
**Lysine**	267.2	108.1	23	265.4	99.4	100	0.866	0.969

Results are expressed as mean (SD). Standardized residuals were examined for outliers, and measurements that were <−3.0 or >3.0 SD from the mean were removed. Adjusted for gestational age. SGA was defined as birth weight below the third percentile.

Supplementary Table 4. Plasma concentrations of amino acids (µmol/L) in preterm infants with or without maternal preeclampsia.

Amino acid	Preeclampsia-yes			Preeclampsia-no			*P*-value	*Adjusted P*-value
	Mean (µmol/L)	SD	N	Mean (µmol/L)	SD	N		
**Glutamate**	117	45	23	127	74	91	0.687	0.162
**Asparagine**	66	23	23	60	24	92	0.187	0.829
**Serine**	173	60	25	160	53	93	0.464	0.373
**Glutamine**	572	250	24	455	177	88	0.084	0.060
**Histidine**	70	19	24	75	20	88	0.221	0.179
**Glycine**	357	153	25	309	112	92	0.318	0.684
**Threonine**	295	103	25	247	94	92	0.115	0.230
**Citrulline**	21	7	25	21	6	91	0.345	0.402
**Arginine**	48	26	25	41	22	93	0.422	0.205
**Alanine**	340	167	22	336	184	90	0.506	0.249
**Taurine**	156	74	24	218	88	94	0.002	0.004
**alpha-Aminobutyric acid**	17	9	25	17	9	94	0.662	0.752
**Tyrosine**	114	43	25	124	46	95	0.348	0.400
**Valine**	154	54	24	184	55	88	0.018	0.029
**Methionine**	50	22	24	59	37	94	0.527	0.262
**Isoleucine**	43	27	24	52	24	90	0.148	0.081
**Phenylalanine**	67	25	24	70	16	89	0.363	0.300
**Tryptophan**	24	8	22	30	9	90	0.005	0.000
**Leucine**	62	19	23	73	25	86	0.178	0.043
**Ornithine**	78	38	23	86	42	91	0.750	0.182
**Lysine**	230	86	24	275	106	91	0.054	0.034

Results are expressed as mean (SD). Standardized residuals were examined for outliers, and measurements that were <−3.0 or >3.0 SD from the mean were removed. Adjusted for gestational age and birth weight.

Supplementary Table 5. Plasma concentrations of amino acids (µmol/L) in preterm infants with or without maternal chorioamnionitis.

Amino acid	Chorioamnionitis-yes			Choriamnionitis-no			*P*-value	*adjusted P*-value
	Mean (µmol/L)	SD	N	Mean (µmol/l)	SD	N		
**Glutamate**	78	56	12	137	72	111	0.007	0.015
**Asparagine**	51	16	12	63	26	111	0.115	0.246
**Serine**	147	28	11	165	57	114	0.956	0.362
**Glutamine**	460	153	12	464	190	106	0.469	0.826
**Histidine**	67	14	12	76	22	108	0.109	0.217
**Glycine**	244	56	11	326	126	111	0.002	0.109
**Threonine**	199	72	12	264	96	111	0.022	0.057
**Citrulline**	18	7	12	21	6	111	0.113	0.114
**Arginine**	55	18	12	41	24	113	0.096	0.055
**Alanine**	243	101	12	345	181	106	0.000	0.110
**Taurine**	160	88	12	215	89	113	0.046	0.031
**alpha-Aminobutyric acid**	16	8	12	17	9	113	0.616	0.658
**Tyrosine**	100	19	12	124	46	114	0.002	0.062
**Valine**	170	25	12	181	62	108	0.027	0.522
**Methionine**	48	34	12	56	30	109	0.261	0.362
**Isoleucine**	47	16	12	50	25	108	0.130	0.620
**Phenylalanine**	71	11	12	69	18	108	0.661	0.626
**Tryptophan**	27	10	12	29	9	106	0.216	0.528
**Leucine**	69	12	11	70	25	103	0.099	0.855
**Ornithine**	67	20	12	89	44	110	0.001	0.140
**Lysine**	217	57	12	272	106	110	0.086	0.097

Results are expressed as mean (SD). Standardized residuals were examined for outliers, and measurements that were <−3.0 or >3.0 SD from the mean were removed. Adjusted for gestational age and birth weight.

Supplementary Table 6. Plasma concentrations of amino acids (µmol/L) in preterm infants exposed or not to antenatal corticosteroids.

Amino acid	Prenatal steroids-yes			Prenatal steroids-no			*P*-value	*adjusted P*-value
	Mean (µmol/L)	SD	N	Mean (µmol/L)	SD	N		
**Glutamate**	130	70	95	120	80	25	0.574	0.302
**Asparagine**	63	26	96	57	22	25	0.628	0.083
**Serine**	169	56	97	144	51	25	0.031	0.041
**Glutamine**	479	196	91	385	139	24	0.124	0.008
**Histidine**	76	21	93	67	20	24	0.272	0.060
**Glycine**	321	111	95	283	141	25	0.062	0.032
**Threonine**	259	96	95	226	66	24	0.165	0.040
**Citrulline**	21	5	91	22	7	26	0.690	0.196
**Arginine**	44	24	97	36	19	25	0.214	0.144
**Alanine**	347	189	92	276	166	24	0.063	0.013
**Taurine**	208	85	97	220	124	26	0.790	0.537
**alpha-Aminobutyric acid**	18	9	97	11	6	26	0.000001	**0.00001**
**Tyrosine**	117	35	93	98	35	23	0.342	0.022
**Valine**	183	59	93	162	52	24	0.469	0.107
**Methionine**	59	36	98	49	31	24	0.977	0.101
**Isoleucine**	51	26	93	42	19	23	0.483	0.147
**Phenylalanine**	69	17	93	66	18	24	0.858	0.453
**Tryptophan**	29	8	91	28	6	22	0.958	0.586
**Leucine**	72	24	89	59	17	23	0.055	0.012
**Ornithine**	87	39	93	72	44	25	0.268	0.056
**Lysine**	274	101	95	218	84	24	0.045	0.009

Results are expressed as mean (SD). Standardized residuals were examined for outliers, and measurements that were <−3.0 or >3.0 SD from the mean were removed. Adjusted for gestational age and birth weight.
